# Essay on Tumors in the Palatine Region

**Published:** 1857-10

**Authors:** 

**Affiliations:** Member of the Anatomical Society, &c.


### ARTICLE II.

*Essay on Tumors in the Palatine Region.* By DR. PARMENTIER, Member of the Anatomical Society, &c. Translated from the French of L'Art Dentaire, for the Am. Jour. Dental Science.

[Continued from the July No., page 339.]

### CHAPTER IV.

*Gummous Tumors.*—Gummous tumors in the vail of the palate have been frequently observed in subjects tainted with constitutional syphilis. It is known that these tumors are among the tertiary symptoms of syphilis. M. Ricord has not said a single word about them, in the notes which he has added to the treatise on syphilis, by Hunter; it is not mentioned in the *Tratié de pathologie externe,* of Vidal, (by Cassis.)

M. Ph. Boyer, who has added a chapter on syphilis to his father's work, only speaks of the ulceration of the velum palati, not mentioning the gummous tumors which have been observed there as well as upon the tongue. MM. Ricord and Vidal have only mentioned them in their *Traité des maladies vêneriennes.* Latterly M. Demarquay has had occasion to observe many gummous tumors of the *velum palati;* I owe to the kindness of MM. Suys, Paupert and Touzelin, the relation of these facts, which will be available in a history of these tumors.

Small and hardly perceptible in the beginning, gummous tumors of the velum palati are hard, adhering to the mucous membrane by a kind of pedicule, and movable under the subjacent and neighboring parts; their growth is slow and without pain, they increase to the size of a small nut, and the mucous which until now had remained without alteration in texture and color became of a brownish blue-red; the submaxillary region tumified, the patient suffered from buzzing in the ears, the deafness becoming sometimes complete; deglutition difficult and painful, and the voice nasal; fluctuation can be perceived through a sort of shell which serves to envelop the tumor; the mucus membrane is perforated in one or more places, and an ichorous pus escapes, not consistent and carrying with it organic debris.

To these perforations shortly succeed an ulceration with projecting borders sharpened to a point and of a grayish color at the base; it progresses rapidly, perforates the vail of the palate, and establishes a communication between the buccal cavity and the posterior part of the nasal fossa. The symptoms of Angina which precede the opening of the tumor were those which attracted the attention of the patient and determined him to have recourse to art; it would be thought upon looking at it that it was simply an abscess of the vail of the palate, if the patient were not questioned concerning his antecedents. The patient of M. Touzelin had had a continual coryza for six months; he had been under the care of M. Hardy for the treatment of rheumatic pain situated in both knees and the left shoulder. The patient of M. Lewis had had previously several large swellings, symptoms which would lead one to suspect an anterior syphilis. If medical aid had been called in the beginning, before there had been any sign of fluctuation, it would have been thought that it was merely a glandular hypertrophy or a cancer. It will be seen later that the tumor caused by glandular hypertrophy, is never adherent to the mucous membrane no matter how old it may be. It has no tendency to ulceration; whereas in a cancerous tumor an entirely different diagnosis can be established. Indeed, we believe that in all cases, it would be well to try the treatment for the tertiary symptoms of syphilis, so as to be perfectly clear in the diagnosis, before deciding to use the knife upon the tumor.

A gummous tumor of the velum palati is always an unhappy prognostic, on account of the disease with which it menaces the patient: I wish to speak of the perforation of the velum palati; this symptom imminently requires a positive diagnosis, and demands very prompt treatment while there is yet time.

One ought, then, to hasten to administer the iodide of potassium, beginning with 7 grammes, (about 12½ grains.) This treatment is proper in all cases, even when there is perforation and ulceration of the velum palati; under its influence, the ulceration very soon deterges itself, and cicatrization begins, the other symptoms of syphilis at the same time disappearing, as was seen by M. Demorquay on the patient concerning whom M. Touzelin spoke to me. The cicatrization of the palatine fistula should be encouraged by repeated cauterizations; but, if not able to effect this, it becomes necessary to perform the operation of staphyloraphy, as was done by M. Demarquay on the patient of M. Paupert.*

*Serous and Hydatid Cysts.*—There does not exist in the annals of science any record of serous cysts of the palatine region: nevertheless it can be readily understood how it is possible for them to be developed in this region; indeed, in the vail of the palate there are several openings from numerous glandular conduits; now one can perceive that these openings being obliterated, the conduit of the glandule is consequently dilated by the accumulation of the secretion and leads to a serous cyst; as to the arch of the palate serous cyst have been already observed for some years on a great number of bones, which is not at all astonishing.

* Thanks to the kindness of M. Demarquay, we hope to be able in the next number, to add to the description of gummous tumors which we have already mentioned, many observations made by this excellent surgeon.—*Fr. Ed.*

Indeed the world is acquainted with the observation of hydatid cysts found in the tonsil reported in the surgical cliniques of Dupytren ; hydatid cysts have been found in several muscles; but no case of hydatid cyst in the palatine region has yet been ɹecorded.

Serous and hydatid cysts should be taken out by the knife.

*Sanguinous Tumors.*—M. Delabarre has observed an aneurism in an artery of the palatine vault; M. Velpeau, who cites the fact in his *Anatomië Chirurgical*, says that if ever such a case presents itself, it would be necessary to treat the disease by the actual cautery, the inequality of the bone and the hardness of the parts preventing the application of the ligature or compress, which have nevertheless been employed with success in hemorrhage of the palatine artery, as I have said in speaking of abcesses.

Scarpa has observed a congenital sanguineous tumor of the palatine arch upon a man aged 47 years, in 1795. The tumor was the size of a chesnut, of a flattish form, the surface was furrowed with points and reddish lines; the general appearance was blackish and bluish. The patient said that he had had the tumor since his earliest childhood, small and then not larger than a pea; it had, with the progress of age, gradually increased in size, and rendered mastication difficult and deglutition excessively painful.

Soft, elastic, painful to the touch, and presenting an enlarged base, this tumor was completely removed by clipping the mucous membrane of the palatine arch with scissors. Blackish, venous blood immediately flowed from the incision in abundance, which was stopped by compression, using a ball of lint saturated with alcohol to which had been added a few drops of sulphuric acid. If a like case occurs it would be well to use perchloride of iron.

The tumor which was extirpated, consisted of a mass of venous vessels closely joined to one another by a thin delicate cellular tissue.

*Second Section.—Solid Tumors.—Hypertrophy of the glandules of the Velum Palati.*—The anatomy of the velum palati shows us the muscles separated from the mucuous membrane by a lamellecular covering, filled with very large follicules, glands which can, in becoming hypertrophied, create tumors which may be likened to the chronic mammary tumors of Astley Cooper to which Prof. Cruveilhier has given the name of *fibrous body of the mammary gland*, and which the microscope has demonstrated, by Prof. Lebert, to be nothing else than a hypertrophy of one or more glandular lobules of the mammary gland. Indeed, this last observer has recognised, in several tumors of the velum palati submitted to his examination, the same elements as in mammary hypertrophy; the microscope has thus confirmed that which could have been foreseen by the scalpel in showing us the identity of aspect of the mammary gland, of the parotid, and of the glandular covering of the velum palati. It has been seen that Prof. Nelaton was the first who called the attention of surgeons to this species of tumor.

Hypertrophied tumors commencing in the superior part of the velum palati develop themselves slowly; the second patient observed by Prof. Nelaton, was 34 years of age, and from the age of 14 he had been aware of the existence of a tumor which had developed itself in the posterior part of the mouth; for some time it remained the size of a hazel nut. The patient operated upon by M. Michon, said that the tumor had appeared about ten years before. It first projected on the interior side of the face; later, in acquiring greater size, it projected from the side of the pharynx, as in the first patient of M. Nelaton, which nevertheless, was not to be observed in the second patient, nor in the patient of M. Michon. The size varies; in this one it was the size of a hen's egg. The mucous membrane which covered the tumor was more deeply colored than in the surrounding parts, was softer and slightly distended. In the second patient of M. Nelaton, a vein, a little enlarged, could be perceived, slightly varicose and running longitudinal; as to the remainder, the mucous membrane is not altered, no matter how old the tumor; it is slippery on the surface, and wrinkles may be made by pinching it—an important symptom to be observed in making up the diagnosis and in operating. The tumors are quite hard, recalling certain fibrous tumors or lymphatic ganglions affected with chronic inflammation; they are clearly marked, and the finger can easily follow all their outlines. They are movable, without adhering to the neighboring tissue and are easily enucleated. These tumors accompany the palate in all its movements, so that one can easily judge of it, if the patient can be induced to make the movement of deglutition, the mouth slightly open so as to allow one to look down into the bottom of the mouth. In developing themselves, these tumors greatly impede deglutition, particularly in liquids; there is the same difficulty in breathing and speaking. Respiration is most difficult at night; speech is muffled and nasal. No matter how old the tumor, the mucous membrane is not likely to ulcerate; ulceration has never been observed in engorgement of the parotid ganglions or inferior maxillaries; the patients never complain of shooting pains there, or any pains whatever. The symptoms we have recounted would easily guide one to make a diagnosis, and would lead to a favorable prognosis.

The functional troubles should determine prompt measures, that is to say the removal of the tumor. The operation does not ordinarily present any great difficulty, for as soon as a vertical incision has been made along the great diameter of the tumor, it can be easily enucleated. It is useless to make any suture to bring together the lips of the wound. Cicatrization ordinanarily answers as well about the twelfth day.

Examined after their removal, these tumors present little lobed projections, rounded and hard, not yielding to a strong pressure, recalling by their consistence, weight and color, the healthy pancreas; for they are of a grayish yellow, sufficiently uniform, presenting a good exterior, through the enveloping membrane as exteriorly upon a fresh cut. If the tumor is opened it does not yield to the cutting without some difficulty, without, however, making the little creaking sound peculiar to scirrhous tumors. The cut has a lobuled aspect; the tissue of the tumor is white, without moisture, covered with fibrous striæ leaving in the intervals a more yellowish tissue, friable, and pierced with numerous little holes ; we find there also, cysts filled with a viscous humor. Finally the microscope has there found hypertrophied culs-de-sac, epithelial cellules but above all epithelial nuclei, some fibro-plastic elements and branches of fibrous tissue.

*Exostosis.*—Recently M. Chassaignac has much insisted upon medio-palatine exostosis as a sign of previous syphilitic affections ; concurring with this surgeon, this sign is of so much importance that it is sometimes the only indication of the previous existence of syphilis and which it is extremely easy to prove.

If the index finger is placed upon the palatine arch, the ball of the finger turned upwards, it comes in contact with a longitudinal protuberance, called by M. Chassaignac *medio-palatine exostosis ;* it is situated in the midst of other abnormal phenomena caused by syphilis more or less remote. In most cases it is only a slight antero-posterior osseous ridge, a species of *veru montanum* of the palate, which very easily escapes observation if one is not prepared for it.

Syphiltic exostosis should be treated by the use of iodide of potassium, in doses of 2, 3, 4 and 5 grammes, commencing with one grain and increasing the dose gradually.

*Osseous Cysts.*—I here recollect the case of Dr. Loire, of an osseous cyst containing a tooth reversed in its interior. A diagnosis of a similar cyst could be made by first examining carefully the superior dental arch, and trying to make the side of the cysts yield, which corresponds to the buccal cavity ; in fact in the case of a serous cyst, one feels the walls yield to a slight pressure of the finger ;* whereas if one had to do with a cyst enclosing a tooth, the tumor would be hard, without elasticity, and it would be impossible to depress it at any point.

*Fibrous Tumors.*—Tumors of this kind, it seems to me, resemble the tumor spoken of by Botot,† the one removed by Anselin,‡ and the one extirpated by Varner.§ The fibrous tumors

* I have corroborated this symptom upon a patient affected with a serous cyst of the inferior maxillary, and which I observed in 1852, under Prof. Malgaigne.

† Jour. de Med. t. xxxvi, p. 269.

‡ Jour. de Med. de Vandermonde, t. xiii, p. 433.

§ Jourdain, Traité des Mal. de la Bouche, t. i, p. 417.

do not accompany the engorgement of lymphatic ganglions and develop themselves slowly. The patient of Anselin had his tumor from his fourteenth or fifteenth year, that of Varner from his seventh year. The fibrous tumors have at all times been seen in the palatine arch, and in developing themselves, greatly interfere with the different functions of the palate: the patient of Varner could only nourish himself with liquids; the one of Anselin could not speak or eat without great difficulty; his respiration, moreover, was always laborious, the tumor being an obstacle to the introduction of air.

These tumors are very hard, Botot says that the tumor of his patient was so hard and tough, chiefly upon its edges, that at each stroke of the bistoury it sounded like the cutting of dried leather. The tumor removed by Varner, seemed to him to have been composed of a cartilaginous substance mixed with long ossiform particles. Their size is variable; nevertheless when patients determine to have the operation performed upon themselves, the tumors do not appear very large, for the tumors in their early stages impede so much the functions of the palatine arch, that those who are affected by them are obliged to have the assistance of art; though in the case before cited, the tumors are not larger than a pigeon's egg.

These tumors should be removed by means of the knife ; but the operation is not without difficulty and danger on account of the hemorrhage which sometimes follows ; the patient of Varner, several hours after the operation, had a hemorrhage which rendered necessary the employment of the actual cautery. In the operation of Anselin, an open artery on the right side, and nearly behind the palate, caused a hemorrhage which required the application of a peculiar compress, contrived by this surgeon for the purpose.

The period taken up in the cure varies; the patient of Botot was cured at the end of three weeks, the one of Anselin in seven, after the exfoliation of several portions of bone.

*Cancerous Tumors.*—These cancerous tumors affect eventually the velum palati as well as the arch; at other times they occupy two parts of the palatine region. The cancer can appear in the beginning in this region, or gradually invade it after having been limited a certain time to the surrounding parts.

In the present state of science, the nature of a tumor should not be decided before it has been examined under the microscope; therefore, one should be careful relative to a tumor similar to the one M. Marjolin removed from the thickness of the vail of the palate, although he has said in his observations upon it, that the tumor was hard, unequal and had the appearance of a cancerous tumor;* these characteristics easily lead one to make an error.

On the contrary the tumor removed from the vault of the palate, in 1844, by Blandin, should be regarded as cancerous, the microscope having demonstrated there, the existence of very marked cancerous cells;† the case was the same in the tumor operated upon in 1849 by M. Chassaignac.‡

Cancerous tumors develop themselves rapidly; the patient operated upon in 1844 by Blandin, was not aware of the tumor until about six weeks before entering the hospital, although the tumor had already grown to the size of five or six centimetres in length, two or three centimetres in thickness in front, at least four behind, and reaching to an height, to the edge of the upper teeth.

These tumors soon give place to engorgement of the neighboring lymphatic ganglions. In the patient that M. Gosselin presented to the Society of Surgery, and upon whom M. Chassaignac operated later, the tumor was eighteen months old, had acquired the size of a small hen's egg, and occupied the right portion of the vail of the palate, the two corresponding pillars, and the tonsil; there was also a line of indurated lumps from the angle of the jaw to the sub-clavicular region. The consistence of these cancerous tumors is sometimes hard, scirrhous, soft, and sometimes elastic (encephaloïd.) These tumors have neither pulse nor fluctuation; ordinarily the patient feels shooting pains there, at other times the tumor is without pain; indeed all the tumors of this region in developing themselves

* Gaz. des Hop. 1851, p. 152.
† Gaz. des Hop. 1844, p. 290, obs. recuillie par M. De Marquay.
‡ Gaz. des Hop. 1849, p. 409.

greatly impede the speech, the digestive functions and respiration.

Cancerous tumors resisting medical treatment should be treated entirely surgically; however, before deciding upon the operation, it is well to attend to the advice of Vidal, (Cassis*) to try in a dose from four to six grammes a day, of iodide of potassium, and if in fifteen days the tumor has not changed, it is necessary to operate immediately.

The success which M. Maisonneuve has obtained with iodide of potassium upon a patient sent by Blandin to Bicetre does not permit us to regard the affection of this patient as being of a cancerous nature, an affection for which M. Blandin contrived an ingenious ligature in 1844.†

### ARTICLE III.

### *American Dental Convention.*

* Gaz. des Hop. 1849, p. 321.

† I again saw the patient at Bicêtre in 1849, he enjoyed perfect health; the engorgement of the soft parts which the isthmus of the throat of this patient presented at the time of his leaving the Hotel-Dieu, had completely disappeared.

